# Supplementary material for: Optogenetic stimulation of Gs-signaling in the heart with high spatio-temporal precision
Source: Nat Commun. 2019 Mar 20;10:1281. doi: 10.1038/s41467-019-09322-7 (PMC6426906; doi:10.1038/s41467-019-09322-7)
Supplement: Supplementary file 2 — Description of Additional Supplementary Files [file 41467_2019_9322_MOESM2_ESM.pdf]

Description of Additional Supplementary Information

File Name: Supplementary Movie 1

Description: Video of a spontaneously beating JellyOp-expressing embryoid body. Light stimulation (309 nW mm-2) is indicated by a blue box in the upper right corner. Video is presented at double speed.
